# Bioaccumulation Capacity of Onion (*Allium cepa* L.) Tested with Heavy Metals in Biofortification

**DOI:** 10.3390/molecules29010101

**Published:** 2023-12-22

**Authors:** Katarzyna Czarnek, Małgorzata Tatarczak-Michalewska, Agnieszka Szopa, Marta Klimek-Szczykutowicz, Karolina Jafernik, Dariusz Majerek, Eliza Blicharska

**Affiliations:** 1 Institute of Medical Science, Faculty of Medical, The John Paul II Catholic University of Lublin, Konstantynów 1 H Str., 20-708 Lublin, Poland; 2 Department of Pathobiochemistry and Interdisciplinary Applications of Ion Chromatography, Biomedical Sciences, Medical University of Lublin, 1 Chodźki Str., 20-093 Lublin, Poland; malgorzatatatarczakmichalewska@umlub.pl; 3 Chair and Department of Pharmaceutical Botany, Jagiellonian University Medical College, Medyczna 9 Str., 30-688 Kraków, Poland; a.szopa@uj.edu.pl (A.S.); karolina.jafernik@doctoral.uj.edu.pl (K.J.); 4 Department of Pharmaceutical Sciences, Collegium Medicum, Jan Kochanowski University, IX Wieków Kielc 19a, 25-516 Kielce, Poland; marta.klimek-szczykutowicz@ujk.edu.pl; 5 Department of Applied Mathematics, Faculty of Mathematics and Information Technology, Lublin University of Technology, Nadbystrzycka 38 Str., 20-618 Lublin, Poland; d.majerek@pollub.pl

**Keywords:** food analysis, bioaccumulation, heavy metals, food safety, toxicity, onion, *Allium cepa* L.

## Abstract

On a worldwide scale, *A. cepa* is among the most commonly consumed vegetables. In Europe, the leading onion producers are Russia, the Netherlands, Spain, Poland and Germany. In this study, the bioaccumulation of heavy metals (Cr, Cu, Zn, Ni, Fe, Mn, Co, Sr, Cd and Pb) by *Allium cepa* L. plants was followed under hydroponic conditions. The heavy metals were applied at six concentrations (0, 25, 50, 100, 200 and 400 mg L^−1^) over three weeks. The quantitative analysis of selected heavy metals in plant tissues (bulbs, roots and assimilation leaves) was performed using atomic absorption spectrometry with flame atomization (F-AAS). The accumulation of metal ions was strongly dependent on their concentrations in the solution and the analyzed parts of plants. The highest accumulation of metal ions was confirmed for the roots and ranged from 8.48 to 5912.34 µg g^−1^ DW (dry weight). All parts of *A. cepa* were characterized by the high accumulation of Mn^2+^. The lowest accumulation was confirmed for Co^2+^ in the roots, Pb^2+^ in the assimilation leaves and Cu^2+^ in the bulbs of onion. Moreover, the study showed that the highest concentrations of heavy metals decreased the growth of bulbs and even caused them to die off. In contrast, lower concentrations of some elements stimulated plant development.

## 1. Introduction

The onion (*Allium cepa* L.) is a biennial plant that belongs to the *Alliaceae* family [1,2,3,4]. On a worldwide scale, *A. cepa* is among the most commonly consumed vegetables after carrots, cabbages, tomatoes and cucumbers [5]. Although it is traditionally grown and consumed, it should be noted that its production has increased by more than 25% in recent years (Food and Agriculture Organization of the United Nations) [6,7]. In Europe, the leading onion producers are Russia, the Netherlands, Spain, Poland and Germany [5,8,9,10].

Owing to their flavor, health-promoting properties and culinary versatility, onions have become a staple in the human diet and cultures over millennia. The edible components of the onion plant, encompassing both green foliage and the bulb, are treasured not only for their culinary applications but also for their nutritional and medicinal properties [11]. *A. cepa* contains phytochemicals such as polyphenols, flavonoids, organic acids, sugars and sulfur compounds, which are beneficial for human health. It is an important vegetable that provides the human body with microelements and vitamins [12,13,14,15].

The presence of vitamins A, B1, B2, C and PP, pectins, hydrocarbons, vegetable proteins, fiber, fat and micro- and macroelements (phosphorus, calcium, magnesium, iron) has been confirmed. In addition, the presence of essential oil has been confirmed, which gives the plant a characteristic odor. The main component of the essential oil is n-propyl disulfide. There are also other sulfur compounds that are alliin homologs, propenylalline and propylalline, which are responsible for the taste and odor of *A. cepa*. When plant tissues are damaged, sulfur compounds break down under the influence of the allinase enzyme, which leads to the formation of thiosulfinic acid derivatives, sulfinyl disulfides, thiophenes and polysulfides. The tear-forming component in onions is propionic thioaldehyde [4,14,16]. Numerous scientific studies have shown that consuming onions reduces the risk of cancer, is anticoagulant and hypoglycemic and lowers blood cholesterol. Volatile and non-volatile sulfur compounds have also been shown to have antibacterial activity. Extracts from the whole plant limit the development of Gram + bacteria of the genera *Streptococcus*, *Staphylococcus*, *Bacillus anthracis* and *Bacillus subtilis* and Gram − bacteria of the genera *Salmonella* and *Shigella* [13,14,17,18].

*A. cepa* is not only a food and medicinal plant. Its properties are also used in environmental monitoring. In many laboratories, it is commonly used to determine toxicity, in the “allium test”. It is used to assess the genotoxicity of various pollutants, e.g., metals, pesticides and aromatic hydrocarbons. Based on the observation of genetic changes that occur in onions under the influence of specific metal ions, their toxicity is assessed. This test also allows the assessment of the mechanisms of action of the examined factors on the DNA of exposed organisms [19,20,21,22,23].

The study of metal accumulation by plants may be utilized in two research areas—soil decontamination and food biofortification. Phytoremediation is the process by which plants effectively remove heavy metals by absorbing them from the contaminated substrate. Some plant species are able to grow on contaminated sites and accumulate large amounts of metals. After absorption, they are either degraded, brought to less harmful forms or transported and accumulated in the various organs. Phytoremediation technologies utilize these hyperaccumulation properties to decontaminate polluted sites [24].

Globally, heavy metals (HMs) are recognized as the most hazardous pollutants, mainly due to their toxicity, ecological risks, non-degradable nature, environmental persistence and biogeochemical recycling nature [25]. Heavy metals in the biogeochemical chain can come from two sources: natural and anthropogenic. Anthropogenic sources include, above all, fuel combustion, waste incineration, non-ferrous metallurgy and iron metallurgy and transport. In uncontaminated soil, metals are commonly found as a result of release from rocks, in soil-forming processes and during volcanic eruptions. Their concentration is not a threat to the ecosystem [26,27].

HMs enter the human body through ingestion or contact with contaminated food, water, air and soil [28]. Some metals, such as iron, zinc, copper, cobalt and manganese, are required for various physiological functions in humans at low concentrations, but they become toxic at higher concentrations. Other HMs, such as cadmium and lead, are not known to have any beneficial effects on human health and their accumulation in the human body is deleterious to health [29]. It is well known that exposure to HMs can cause hemopoietic, cardiovascular, gastrointestinal, respiratory, reproductive, renal and neurological disorders [30,31]. Among the most common environmental and industrial HMs, cadmium, nickel and hexavalent chromium are classified as Group 1 carcinogens by the International Agency for Research on Cancer (IARC) [32]. Exposure to them is strongly associated with an increased risk of various types of cancer, such as GI cancers, lung cancer and breast cancer [33,34,35,36,37,38]. Multiple mechanisms have been discovered to contribute to HM-induced carcinogenesis, including abnormal signaling transduction, oxidative stress and DNA damage and repair. Alterations of epigenetic modulations, such as DNA methylation, histone modification and non-coding RNA, have also been shown to contribute to metal carcinogen-induced cell transformation [33,39,40].

About one third of the world’s population is affected by concealed hunger or malnutrition owing to micronutrient deficiencies. Current food production is not sufficient to feed the growing population, and what is provided lacks micronutrients. Globally, Fe deficiency is the most widespread nutritional disorder and affects approximately two billion people. In Europe, malnutrition problems related to diets with low micronutrient content are increasing the number of children and women with anemia. Zinc is the second-highest micronutrient deficiency, affecting approximately 17% of the global population. Therefore, the need to address malnutrition with improved food quality has arisen. In the case of non-processed food, such as vegetables, the only option to enhance the nutrient content of products is in preharvest, using improved genotypes or adopting specific agronomical techniques [41,42,43]. Biofortification is the process of increasing the concentrations and bioavailability of essential nutrients in a staple crop through traditional plant breeding, agronomic practices and/or genetic engineering; it is a potential approach to combat micronutrient deficiencies at the population level [44].

Nowadays, hydroponic cultivation is gaining more and more popularity all over the world thanks to efficient resource management and the production of high-quality food. Growing food in a traditional way, i.e., in soil, has various problems, such as significant urbanization, natural disasters, climate change and the massive use of chemicals and pesticides, which reduces soil fertility. This all affects the significant development of hydroponics. The advantages of this method are primarily shorter cultivation times compared to the conventional method, plant production throughout the year and the minimal occurrence of diseases and pests; we can also eliminate the weeding, spraying and watering of plants. The NFT technique is used all over the world, primarily to grow leafy vegetables. The NFT technique allows water savings from 70 percent to 90 percent. The leading countries in hydroponic technology are the Netherlands, France, Australia, Israel, England, Canada and the USA [45,46,47]. Researchers have explored the applications of this cultivation technique to reveal its future importance. This technique can be adapted to almost all terrestrial plants. Vegetable food crops like wheat, tomato, marijuana, dill and many more plants are being cultivated at a commercial scale. The construction of a hydroponic system requires an initial investment, hard work and care. It is recommended that this technique is adapted to produce food crops and medicinal plants to meet the global demand, to control global warming and thereby preserve the Earth for a better future [47,48].

Since *A. cepa* is one of the most frequently cultivated and consumed vegetables, the aim of the present work was to evaluate the accumulation ability of selected heavy metals in plant tissues (bulbs, roots and assimilation leaves). *A. cepa* var. Wolska underwent biofortification with Cr, Cu, Zn, Ni, Fe, Mn, Co, Sr, Cd and Pb solutions in hydroponic media at six concentrations in the range of 0 to 400 mg L^−1^ over three weeks. The quantitative analysis of heavy metals in the plant tissues was performed using atomic absorption spectrometry with flame atomization. Moreover, the experiment evaluated the effect of the toxicity of metal ions and their concentrations on the appearance of onion growing in different solutions over a time period of three weeks. The obtained results and observations will contribute to the estimation of the possibility of using *A. cepa* as a phytoremediator; determining the possibility of using biofortification, i.e., enriching *A. cepa* with some elements necessary in the diet; and determining the potential risk of growing onions on soil contaminated with heavy metals.

## 2. Results

### 2.1. Morphological Analysis

The experiment evaluated the effect of the toxicity of metal ions and their concentrations on the appearance of *A. cepa* growing on different solutions over a time period of three weeks. The study showed that the highest concentrations of heavy metals decreased the growth of bulbs and even caused them to die off. In contrast, lower concentrations of some elements stimulated plant development. The harmful effect of the metals was dependent on the developmental stage of the *A. cepa* studied.

In the control sample, we observed rapid root development. After 10 days, assimilation leaves appeared. The plant developed properly. A change in the color of leaves was not observed.

**Figure 1:**
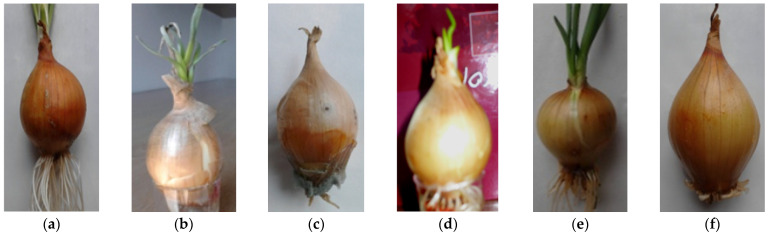
Morphological appearance of *A. cepa* grown in hydroponics supplemented with different concentrations of metal ions: 400 mg L^−1^ Sr^2+^ (**a**), 400 mg L^−1^ Ni^2+^ (**b**), 50 mg L^−1^ Cr^3+^ (**c**), 400 mg L^−1^ Zn^2+^ (**d**), 100 mg L^−1^ Pb^2+^ (**e**), 200 mg L^−1^ Cd^2+^ (**f**).

The plants growing on solutions with different concentrations of Mn^2+^, Fe^3+^ and Sr^2+^, even at the highest concentrations, did not show growth inhibition of the aerial part. Characteristic of these variants was the stimulation of increased roots (Figure 1a). In comparison to the control samples, in these variants, faster growth was observed.

Differences in plant growth were confirmed on solutions with Co^2+^, Cu^2+^ and Ni^2+^. At the concentrations of 25 and 50 mg L^−1^, the smaller growth of tubers was observed compared to control samples. At the higher concentrations, after assimilation leaves appeared, their darkening and drying out was observed (Figure 1b). Meanwhile, the roots were dying.

The low concentrations of Cr^3+^ in the solution caused the slowed growth of assimilation leaves and roots compared to controls, and the roots began to rot. A reduction in the length of the roots and even growing decay were caused by disturbances in cell division and growth cones. The leaves withered and changed color. The concentration of 50 mg L^−1^ completely inhibited plant growth (Figure 1c).

The normal development of *A. cepa* at the concentrations of 25, 50 and 100 mg L^−1^ Zn^2+^ ions in solutions in comparison to the control samples was observed. After two weeks, the formation of assimilation leaves occurred. A high concentration of zinc caused morphological changes—the shortening of roots and the weaker development of assimilation leaves (Figure 1d).

The plants grown in solutions containing Pb^2+^ were characterized by a reduced mass and amount of roots compared to control samples. At a concentration of 100 mg L^−1^, *A. cepa* possessed high tolerance compared to other variants (Figure 1e).

The toxic effects of Cd^2+^ on plant development have already been observed above 25 mg L^−1^. Higher concentrations of Cd^2+^ in the solutions were characterized by reduced amounts of roots, which was caused by their dying. The root analysis showed a reduction in their length depending on the increased concentration of Cd^2+^ ions in the solution. At concentrations from 50 to 400 mg L^−1^, the quantitative analysis was impossible due to the lack of roots. Under these concentration conditions, assimilation leaf formation was also inhibited (Figure 1f).

### 2.2. Accumulation of Metal Ions

**Figure 2:**
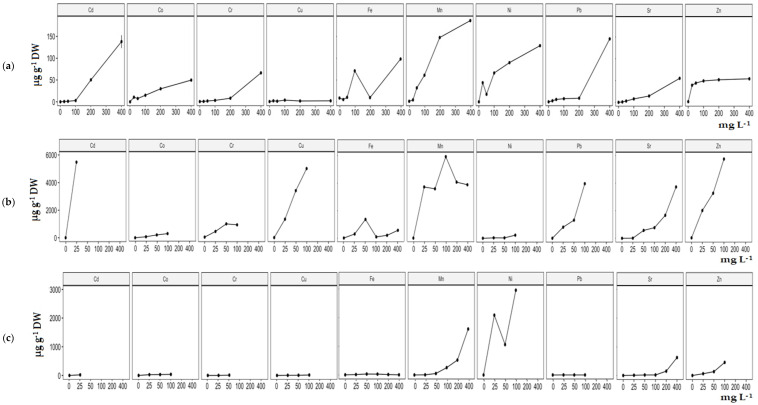
Dependence of the content of metal ions (µg g^−1^ DW) in bulbs (**a**), roots (**b**) and assimilation leaves (**c**) of *A. cepa* on the concentration of metal ions in the solution (mg L^−1^).

The accumulation of metal ions was strongly dependent on their concentration in the solution and the analyzed parts of the plants (Figure 2, Table 1, Table 2, Table 3 and Tables S1–S10). The higher accumulation of metal ions was confirmed for the roots and ranged from 8.48 to 5912.34 µg g^−1^ DW (Table 1). In bulbs and assimilation leaves, the content of metal ions was dependent on the tested metal ions and their concentration in the solution. Assimilation leaves were characterized by highly varied metal ion content, from 0.70 to 1600.92 µg g^−1^ DW (Table 2). The content of accumulated metal ions in the bulbs of *A. cepa* ranged from 0.60 to 186.38 µg g^−1^ DW (Table 3). All parts of *A. cepa* were characterized by the high accumulation of Mn^2+^ (Table 1, Table 2, Table 3 and Tables S1–S10). The lowest accumulation was confirmed for Co^2+^ in the roots, Pb^2+^ in the assimilation leaves and Cu^2+^ in the bulbs of *A. cepa* (Table 1, Table 2, Table 3 and Tables S1–S10).

The research also included an analysis of metal ions in the control sample. In the roots and bulbs, for the following metal ions, the maximal content was obtained in the roots—Cr^3+^ (max. 47.01 µg g^−1^ DW), Zn^2+^ (max. 30.41 µg g^−1^ DW), Fe^3+^(max. 16.81 µg g^−1^ DW), Mn^2+^ (max. 11.65 µg g^−1^ DW) and Cu^2+^ (max. 10.88 µg g^−1^ DW) (Table 1 and Table 3). In the assimilation leaves, only two metal ions were detected, namely Fe^3+^ (4.74 µg g^−1^ DW) and Mn^2+^ (0.64 µg g^−1^ DW) (Table 2).

In all parts of *A. cepa*, the high content of Mn^2+^ among other metal ions was confirmed. Mn^2+^ were the ions with the largest differences in accumulation (Table 1, Table 2, Table 3 and Table S1). The content of Mn^2+^ ranged in the roots from 3589.09 to 5912.34 µg g^−1^ DW, in the assimilation leaves from 5.16 to 1600.92 µg g^−1^ DW and in the bulbs from 4.49 to 186.38 µg g^−1^ DW (Table 1, Table 2, Table 3 and Table S1). In the assimilation leaves and bulbs, the content of Mn^2+^ increased with the increasing concentration of these ions in the solution; the maximum value was obtained for a concentration in the solution of 400 mg L^−1^ (Table 2, Table 3 and Table S1). In the roots, the highest accumulation was obtained for 100 mg L^−1^. At concentrations of 200 and 400 mg L^−1^, a decrease in the content of these ions was characteristic (Table S1). The maximum Mn^2+^ content (5912.34 µg g^−1^ DW) was obtained in the roots grown on a solution with 100 mg L^−1^ metal ions, and the lowest (4.49 µg g^−1^ DW) in the bulbs in a solution with a concentration of 25 mg L^−1^ (Table 1, Table 2, Table 3 and Table S1).

In the roots and assimilation leaves, the amount of Zn^2+^ depended on the concentration of these ions in the solution. In the bulbs, a difference between the concentration and content was not observed. The content of Zn^2+^ ranged from 2015.34 to 5743.33 µg g^−1^ DW in the roots, from 62.34 to 457.06 µg g^−1^ DW in the assimilation leaves and from 39.21 to 53.97 µg g^−1^ DW in the bulbs (Table 1, Table 2, Table 3 and Table S5). In all parts, the lowest accumulation was confirmed at a solution concentration of 25 mg L^−1^ (Table S5). In the roots and assimilation leaves, the highest accumulation was obtained for 100 mg L^−1^, and in the bulbs for a concentration of 400 mg L^−1^ (Table 1, Table 2, Table 3 and Table S5). In view of the inhibition of root growth and assimilation leaves, the content for 200 and 400 mg L^−1^ was not evaluated. The maximum Zn^2+^ content (5743.33 µg g^−1^ DW) was obtained in the roots grown on a solution with 100 mg L^−1^ metal ions, and the lowest (39.21 µg g^−1^ DW) in the bulbs in a solution with a concentration of 25 mg L^−1^ (Table 1, Table 2, Table 3 and Table S5).

Large differences between all parts of *A. cepa* in terms of the content of Cu^2+^ were confirmed (Table 1, Table 2, Table 3 and Table S10). The content of Cu^2+^ ranged from 1347.66 to 5013.33 µg g^−1^ DW in the roots, from 4.74 to 14.28 µg g^−1^ DW in the assimilation leaves and from 1.37 to 3.86 µg g^−1^ DW in the bulbs (Table 1, Table 2, Table 3 and Table S10). In the assimilation leaves and roots, the content of Cu^2+^ was higher with an increasing concentration of these ions in the solution, and the maximum value was obtained for a solution concentration of 100 mg L^−1^ (Table 1, Table 2 and Table S10). In the bulbs, the higher accumulation was obtained also for 100 mg L^−1^, but the accumulation did not depend on the increasing metal ion concentrations. In view of the inhibition of root growth and assimilation leaves, the content for 200 and 400 mg L^−1^ was not evaluated (Table S10). The maximum Cu^2+^ content (5013.33 µg g^−1^ DW) was obtained in the roots grown in a solution with 100 mg L^−1^ metal ions, and the lowest (1.38 µg g^−1^ DW) in the storage plants in a solution with a concentration of 50 mg L^−1^ (Table 1, Table 2, Table 3 and Table S10). Cu^2+^ ions were clearly accumulated in the roots.

For Pb^2+^, in the bulbs and roots, the content of ions depended on the concentration in the solution. In the assimilation leaves, differences between the concentrations and content were not observed. The content of Pb^2+^ ranged from 805.10 to 3961.00 µg g^−1^ DW in the roots, from 0.70 to 1.17 µg g^−1^ DW in the assimilation leaves and from 2.40 to 144.47 µg g^−1^ DW in the bulbs (Table 1, Table 2, Table 3 and Table S3). In the bulbs and roots, the lowest accumulation was confirmed at a solution concentration of 25 mg L^−1^ (Table S3) and in the assimilation leaves at 50 mg L^−1^. In the roots, the highest accumulation was obtained for 100 mg L^−1^, in the bulbs at a concentration of 400 mg L^−1^ and in the assimilation leaves at a concentration of 25 mg L^−1^ (Table 1, Table 2, Table 3 and Table S3). In view of the inhibition of root growth and assimilation leaves, the content for 200 and 400 mg L^−1^ was not evaluated. The maximum Pb^2+^ content (3961.00 µg g^−1^ DW) was obtained in the roots grown on a solution with 100 mg L^−1^ metal ions, and the lowest (0.70 µg g^−1^ DW) in the assimilation leaves on a solution with a concentration of 50 mg L^−1^ (Table 1, Table 2, Table 3 and Table S3).

In all parts of *A. cepa*, the content of Sr^2+^ was higher with an increasing concentration of ions in the solution (Table 1, Table 2, Table 3 and Table S6). The content of Sr^2+^ ranged from 8.48 to 3727.27 µg g^−1^ DW in the roots, from 9.42 to 626.64 µg g^−1^ DW in the assimilation leaves and from 0.93 to 55.07 µg g^−1^ DW in the bulbs (Table 1, Table 2, Table 3 and Table S6). In all parts of *A. cepa*, the maximal values were obtained for a solution concentration of 400 mg L^−1^, and the lowest at the concentration of 25 mg L^−1^ (Table 1, Table 2, Table 3 and Table S6). The maximum Sr^2+^ content (3727.27 µg g^−1^ DW) was obtained in the roots grown on a solution with 400 mg L^−1^ metal ions, and the lowest (0.93 µg g^−1^ DW) in the bulbs in a solution with a concentration of 25 mg L^−1^ (Table 1, Table 2, Table 3 and Table S6).

The content of Ni^2+^ ranged in the roots from 1057.85 to 2956.00 µg g^−1^ DW, in the assimilation leaves from 27.94 to 224.70 µg g^−1^ DW and in the bulbs from 17.38 to 128.82 µg g^−1^ DW (Table 1, Table 2, Table 3 and Table S2). In all parts of *A. cepa*, the lowest accumulation was confirmed in the solution of concentration of 50 mg L^−1^ (Table S2). In the roots and assimilation leaves, the highest accumulation was obtained for 100 mg L^−1^, and in the bulbs at a concentration of 400 mg L^−1^ (Table 1, Table 2, Table 3 and Table S2). In view of the inhibition of root growth and assimilation leaves, the content for 200 and 400 mg L^−1^ was not evaluated. The maximum Ni^2+^ content (2956.00 µg g^−1^ DW) was obtained in the roots grown in a solution with 100 mg L^−1^ metal ions, and the lowest (17.38 µg g^−1^ DW) in the bulbs in a solution with a concentration of 50 mg L^−1^ (Table 1, Table 2, Table 3 and Table S2).

In all parts of *A. cepa*, a dependence between the content of Fe^3+^ and concentration was not confirmed (Table 1, Table 2, Table 3 and Table S8). The content of Fe^3+^ ranged from 97.79 to 1370.80 µg g^−1^ DW in the roots, from 5.46 to 33.49 µg g^−1^ DW in the assimilation leaves and from 5.26 to 98.25 µg g^−1^ DW in the bulbs (Table 1, Table 2, Table 3 and Table S8). The lowest accumulation was confirmed in the roots at a solution concentration of 100 mg L^−1^, in the assimilation leaves at 400 mg L^−1^ and in the bulbs at 25 mg L^−1^ (Table 1, Table 2, Table 3 and Table S8). In the roots and assimilation leaves, the highest accumulation was obtained for 50 mg L^−1^, and in bulbs at 400 mg L^−1^ (Table 1, Table 2, Table 3 and Table S8). The maximum Fe^3+^ content (1370.80 µg g^−1^ DW) was obtained in the roots grown in a solution with 50 mg L^−1^ metal ions, and the lowest (5.26 µg g^−1^ DW) in the bulbs in a solution with a concentration of 25 mg L^−1^ (Table 1, Table 2, Table 3 and Table S8).

For Cr^3+^, in the bulbs and assimilation leaves, the content of ions was dependent on the concentrations in the solutions. In the roots, a dependence between the concentrations and content was not observed. The content of Cr^3+^ ranged from 459.25 to 1008.42 µg g^−1^ DW in the roots, from 0.88 to 9.56 µg g^−1^ DW in the assimilation leaves and from 0.92 to 66.37 µg g^−1^ DW in the bulbs (Table 1, Table 2, Table 3 and Table S7). In the roots, bulbs and assimilation leaves, the lowest accumulation was confirmed at a solution concentration of 25 mg L^−1^ (Table S7). In the roots and assimilation leaves, the highest accumulation was obtained for 50 mg L^−1^, and in the bulbs at a concentration of 400 mg L^−1^ (Table 1, Table 2, Table 3 and Table S7). In view of the inhibition of root growth and assimilation leaves, the content for 100, 200 and 400 mg L^−1^ for assimilation leaves and 200 and 400 mg L^−1^ for roots was not evaluated. The maximum Cr^3+^ content (1008.42 µg g^−1^ DW) was obtained in the roots grown in a solution with 50 mg L^−1^ metal ions, and the lowest (0.88 µg g^−1^ DW) in the assimilation leaves in a solution with a concentration of 25 mg L^−1^ (Table 1, Table 2, Table 3 and Table S7).

For Co^2+^, its accumulation in *A. cepa* was lower compared to the abovementioned metal ions. In the roots and assimilation leaves, the amount of Co^2+^ was dependent on the concentration of these ions in the solutions. The content of Co^2+^ ranged from 71.80 to 302.49 µg g^−1^ DW in the roots, from 25.86 to 37.77 µg g^−1^ DW in the assimilation leaves and from 7.51 to 49.79 µg g^−1^ DW in the bulbs (Table 1, Table 2, Table 3 and Table S9). The lowest accumulation in the roots and assimilation leaves was confirmed at a solution concentration of 25 mg L^−1^, and for bulbs of 50 mg L^−1^ (Table S9). In the roots and assimilation leaves, the highest accumulation was obtained for 100 mg L^−1^, while, in the storage part, it was observed in a concentration of 400 mg L^−1^ (Table 1, Table 2, Table 3 and Table S9). In view of the inhibition of root growth and assimilation leaves, the content for 200 and 400 mg L^−1^ was not evaluated. The maximum Co^2+^ content (302.49 µg g^−1^ DW) was obtained in the roots grown in a solution with 100 mg L^−1^ metal ions, and the lowest (7.51 µg g^−1^ DW) in the bulbs on a solution with a concentration of 50 mg L^−1^ (Table 1, Table 2, Table 3 and Table S9).

In view of the inhibition of root growth and assimilation leaves in *A. cepa* grown in a solution with Cd^2+^, for this part, only the concentration of 25 mg L^−1^ was analyzed (Table 1, Table 2 and Table S4). In bulbs, the content was dependent on the concentration of Cd^2+^ ions in the solution and ranged from 0.60 to 138.08 µg g^−1^ DW. The maximal content in this part was at a concentration of 400 mg L^−1^ and the lowest at 25 mg L^−1^ (Table 3). The roots grown in low concentrations at a high level accumulated Cd^2+^, and the content of these ions in the roots in a solution of 25 mg L^−1^ was equal to 5478.00 µg g^−1^ DW (Table 1). In the assimilation leaves, at a concentration of 25 mg L^−1^, the content was 20.03 µg g^−1^ DW (Table 2).

## 3. Discussion

The presented study documents, for the first time, in the roots, bulbs and assimilation leaves of *A. cepa*, the accumulation of different metal ions and their concentrations. The research also focused on the plant’s appearance during the accumulation of these metal ions. In control samples grown in a solution without the tested metal ions, we confirmed the presence of the following metal ions: Cr^3+^, Cu^2+^, Fe^3+^, Mn^2+^ and Zn^2+^ for roots and bulbs; Mn^2+^ and Fe^3+^ for assimilation leaves. The maximal content of Cr^3+^, Cu^2+^, Fe^3+^ Mn^2+^ and Zn^2+^ in roots and bulbs grown in a solution containing the tested metal ions was, respectively, 21.5, 460.8, 81.5, 507.5 and 188.9 times higher for roots and 122.9, 7.6, 11.3, 154.0 and 35.5 times higher for bulbs than in control samples (Table 1 and Table 3). The maximal content of Mn^2+^ and Fe^3+^ in the assimilation leaves grown in a solution containing the tested metal ions was, respectively, 2501.4 and 7.1 times higher than in control samples (Table 2).

The studies confirmed that the maximal accumulation of metal ions increased in the following order for *A. cepa* roots: Co^2+^ < Cr^3+^ < Fe^3+^ < Ni^2+^ < Sr^2+^ < Pb^2+^ < Cu^2+^ < Cd^2+^ < Zn^2+^ < Mn^2+^. For assimilation leaves, it was Pb^2+^ < Cr^3+^ < Cu^2+^ < Cd^2+^ < Fe^3+^ < Co^2+^ < Ni^2+^ < Zn^2+^ < Sr^2+^ < Mn^2+^; for bulbs, it was Cu^2+^ < Co^2+^ < Zn^2+^ < Sr^2+^ < Cr^3+^ < Fe^3+^ < Ni^2+^ < Cd^2+^ < Pb^2+^ < Mn^2+^ (Table 1, Table 2 and Table 3).

Chromium accumulation was also the focus of the studies by Zayed et al. [49]. They studied the accumulation of two ions of chromium in different species, such as *Brassica oleracea* L. var. *capitata* L. (cabbage), *B. oleracea* L. var. *botrytis* L. (cauliflower), *Apium graveolens* L. var. *dulce* (Mill.) Pers. (celery), *Allium schoenoprasum* L. (chive), *B. oleracea* L. var. *acephala* DC. (collard), *Pisum sativum* L. (garden pea), *B. oleracea* L. var. *acephala* DC. (kale), *Lactuca sativa* L. (lettuce), *A. cepa* L. (onion), *Spinacia oleracea* L. (spinach) and *Fragaria × ananasa* Duch. (strawberry). The plants were cultured hydroponically using half-Hoagland’s solution. After one week, the plants were supplied with chromium in two forms: one group of plants received 1 mg L^−1^ Cr^6+^ supplied as potassium chromate, and the other group of plants received 1 mg L^−1^ Cr^3+^ added as chromium chloride. After one week with chromium treatment, they were harvested. In this study, for all plants, the higher accumulation of Cr ions in the roots compared to the shoots was confirmed. This result was also observed in our studies. *A. cepa* was characterized by the smallest accumulation compared to another species. The Cr ion concentrations in the roots and shoots did not exceed 30 and 1 mg kg^−1^ DW, respectively. Our results confirmed the increasing accumulation in the roots as the concentration increased. The maximal value (1008.42 mg kg^−1^ DW) of growth was obtained at a concentration of 50 mg L^−1^ after 3 weeks, and it was 33.6 times higher than in the results obtained by Zayed et al. [49] at a concentration of 1 mg L^−1^ Cr ions after 1 week of culture (Table 1 and Table S7). For *B. oleracea* L. var. *capitata* L., *B. oleracea* L. var. *botrytis* L. and *B. oleracea* L. var. *acephala* DC grown in a solution with 1 mg L^−1^ Cr ions, the high accumulation of ions in roots was detected (more than 350 mg kg^−1^ DW). It was only 1.3 times lower than in *A. cepa* cultured in a solution containing 25 mg L^−1^ Cr ions (459.25 mg kg^−1^ DW) (Table 1 and Table S7). This indicates the higher accumulation of Cr ions in these species [49].

Another study focused on the accumulation of Pb^2+^ in different species, such as *Biscutella laevigata* (buckler mustard), *Brassica napus* (rapeseed), *Cucumis sativus* (cucumber), *Hordeum vulgare* (barley), *Leontodon hispidus* (bristly hawkbit), *Lupinus luteus* (lupine), *Phaseolus vulgaris* (bean), *Pisum sativum* (pea), *Raphanus sativus* (radish), *Secale cereale* (rye), *Silene vulgaris*, *Soja hispida* (soy bean), *Triticum vulgare* (wheat) and *Zea mays* (maize), and also several cultivars of *A. cepa*, namely *A. cepa* var. Kutnowska, *A. cepa* var. Sochaczewska and *A. cepa* var. Wolska [50]. The plants were grown on 1/8 Knop’s medium containing PbCl_2_ in a concentration of 5 mg L^−1^ Pb^2+^ ions and incubated for 7 days. For all species, accumulation was higher in the roots than in the shoots. The accumulation of Pb^2+^ in *A. cepa* var. Wolska in this study was the lowest compared to other *A. cepa* cultivars. At a concentration of 5 mg L^−1^ Pb^2+^, after 7 days of cultivation, *A. cepa* var Wolska accumulated 9046 mg kg^−1^ DW in the roots and 1043 mg kg^−1^ in the shoots. For the roots, it was 2.3 times higher than in our maximum results for *A. cepa* grown in a solution containing 100 mg L^−1^ Pb^2+^ (3961 mg kg^−1^) (Table 1 and Table S3). For the bulbs, they obtained about seven times higher accumulation than in our results. Other tested species were characterized by the significantly higher accumulation of Pb^2+^ in comparison to our results. The highest accumulation in the roots was confirmed for *B. napus* (55,680 mg kg^−1^ DW); it was 14.1 times higher than in our result for *A. cepa* roots (Table 1 and Table S3). For shoots, the maximal accumulation was obtained in *C. sativus* (2095 mg kg^−1^ DW) and it was 14.5 times higher compared to the bulbs of *A. cepa* (Table 1 and Table S3) [50].

The accumulation of cadmium, copper, lead and zinc in *Polygonum thunbergii* was the focus of the studies by Kim et al. [51]. *P. thunbergii* was hydroponically grown for 6 days in modified Hoagland’s solution containing one of the tested metal ions in concentrations of 44 mg L^−1^ for Cd^2+^, 82 mg L^−1^ for Pb^2+^, 26 mg L^−1^ for Cu^2+^ and 25 mg L^−1^ for Zn^2+^. The content of metal ions was evaluated every 24 h. The accumulation by *P. thunbergii* increased in the order of cadmium < lead < zinc < copper. In *A. cepa*, for the roots, the accumulation was characterized by a different sequence of lead < copper < cadmium < zinc (Table 1). This confirms the differences in accumulation by different species. The maximal content of metal ions in *P. thunbergii* was obtained after 6 days of cultivation. The maximal amounts of Cd^2+^ (87.7 µg g^−1^ DW), Cu^2+^ (450.2 µg g^−1^ DW), Pb^2+^ (263.1 µg g^−1^ DW) and Zn^2+^ (333.7 µg g^−1^ DW) for *P. thunbergii* were, respectively, 62.5, 3.0, 5.0 and 6.0 times lower than in the roots of *A. cepa* and 146.2, 202.8, 48.2 and 8.5 times higher than in the bulbs of *A. cepa* grown in a solution containing 25 mg L^−1^ Cd^2+^, 25 mg L Cu^2+^, 50 mg L^−1^ Pb^2+^ and 25 mg L^−1^ Zn^2+^ (Tables S3–S5 and S10) [51].

The accumulation of cadmium was also the focus of the research by Solis-Dominguez et al. [52]. They investigated the accumulation of Cd^2+^ in *Echinochloa polystachya* grown for 58 days in Long Ashton nutrient solution containing seven different concentrations of Cd^2+^ (0, 0.25, 1, 2, 10, 50 and 100 mg L^−1^). The highest Cd^2+^ accumulation was recorded within the plant subjected to the highest concentration—100 mg L^−1^. The maximal content of Cd^2+^ in the roots and leaves was 299 and 233 mg kg^−1^ DW, respectively, at a concentration of 100 mg L^−1^, and it was 18.3 times lower and 11.6 times higher, respectively, than in the roots and assimilation leaves of *A. cepa* grown in a solution containing 25 mg L^−1^ Cd^2+^ (Table 1 and Table 2) [52].

Another study focused on the accumulation of Cd^2+^ and Pb^2+^ in four plants, sunflower (*Helianthus annuus* L.), mustard (*Brassica juncea* L.), alfalfa (*Medicago sativa* L.) and ricinus (*Ricinus communis* L.), growing hydroponically for 5 weeks in Hoagland’s solution containing different concentrations of metal ions: Cd^2+^—5, 10, 20 mg L^−1^ and Pb^2+^—50, 100 and 200 mg L^−1^ [53]. The roots and aerial parts were completely analyzed. The highest accumulation was confirmed for *H. annus* (327.34 mg kg^−1^ DW) in a solution with 20 mg L^−1^ Cd^2+^ and it was 16.7 times lower than in *A. cepa* roots grown in a solution containing 25 mg L^−1^ Cd^2+^ (Table 1). For Pb^2+^, the highest accumulation (917.82 mg kg^−1^ DW) was obtained also for *H. annus* in a solution with 200 mg L^−1^ Pb^2+^, and it was 4.3 times lower than in *A. cepa* roots grown in a solution containing 100 mg L^−1^ Pb^2+^ (Table 1) [53].

Another study showed the accumulation of Mn^2+^ in *Phytolacca americana* [54]. The whole plants were grown for 12 days in solutions with different concentrations of Mn^2+^. The tested concentrations were 5.49, 54.94, 137.35, 274.70, 549.38, 1373.45 and 2746.90 mg L^−1^ of Mn^2+^ added to Hoagland’s nutrient solution. The analyzed plant material was the leaves, stems and roots. The maximal content for these ions in leaves (2,292,000 µg g^−1^ DW), roots (643,000 µg g^−1^ DW) and stems (501,000 µg g^−1^ DW) was confirmed in a solution containing 549.38 mg L^−1^; at higher concentrations, the content decreased. The maximal accumulation for *P. americana* roots was 387.7 times higher than in *A. cepa* roots grown in a solution with 400 mg L^−1^ Mn^2+^ (Table 1 and Table S1) [54].

The accumulation of Cd^2+^, Co^2+^, Cu^2+^ and Ni^2+^ in *Allium sativum* L. (garlic) was the object of the research by Soudek et al. [55]. The bulbs of *A. sativum* were grown in Erlenmeyer flasks containing solutions with different metal ions dissolved in distilled water for 14 days. The analyzed plant material was the roots, bulbs and leaves. For each metal ion, they tested two concentrations (50 and 250 µmol) for Cd^2+^ (5.62 and 28.1 mg L^−1^), Co^2+^ (2.95 and 14.73 mg L^−1^), Cu^2+^ (3.18 and 15.89 mg L^−1^) and Ni^2+^ (2.93 and 14.67 mg L^−1^). For all tested metal ions except Co^2+^, at a concentration of 14.73 mg L^−1^, the accumulation in different parts of *A. sativum* increased in the order of leaves < bulbs < roots. In our results for the accumulation of this metal ion at a concentration of 25 mg L^−1^ in different parts of *A. cepa*, we obtained this sequence only for Ni^2+^. In the other metal ions, the accumulation increased in the order of bulbs < leaves < roots (Tables S2, S4, S9 and S10). For roots, the maximal content of accumulated Cd^2+^ (1830 µg g^−1^ DW), Ni^2+^(820 µg g^−1^ DW) and Cu^2+^ (630 µg g^−1^ DW) was, respectively, 3.0, 2.5 and 2.1 times lower than in *A. cepa* roots grown at a concentration of 25 mg L^−1^ for this metal ion (Tables S2, S4, S9 and S10). Only the maximal content of Co^2+^ in *A. sativum* roots was characterized by 11.3 times higher accumulation compared to *A. cepa* roots (Tables S2, S4, S9 and S10). The maximal accumulation in *A. sativum* bulbs of Cd^2+^ (1120 µg g^−1^ DW), Ni^2+^(240 µg g^−1^ DW), Cu^2+^ (210 µg g^−1^ DW) and Co^2+^ (160 µg g^−1^ DW) was, respectively, 1866.7, 5.5, 94.5 and 15.2 times higher than in *A. cepa* bulbs grown at a concentration of 25 mg L^−1^ of the tested metal ions. For leaves, *A. sativum* was also characterized by significantly higher accumulation; in this case, the content of Cd^2+^ (310 µg g^−1^ DW), Ni^2+^(250 µg g^−1^ DW), Co^2+^ (190 µg g^−1^ DW) and Cu^2+^ (30 µg g^−1^ DW) was, respectively, 15.5, 8.3, 7.3 and 6.3 times higher compared to *A. cepa* leaves grown at a concentration of 25 mg L^−1^ of the tested metal ions (Tables S2, S4, S9 and S10). The results confirmed the higher accumulation of Cd^2+^, Cu^2+^ and Ni^2+^ in the roots of *A. cepa* and lower in other parts in comparison to *A. sativum*. For Co^2+^, its accumulation in all parts of *A. sativum* was higher than in *A. cepa* (Tables S2, S4, S9 and S10) [55].

## 4. Materials and Methods

### 4.1. Plant Material

The studied materials were *A. cepa* cv. ‘Wolska’ bulbs, which were obtained from the country’s manufacturer. The bulbs were grown without pesticides and met nutrition requirements. They had an average height of 4 cm and mass of about 35–50 g. Before the experiment, the bulbs was cleaned and weighed.

During the experiment, the bulbs were maintained on different variants of metal salt solutions. The following metal ions were tested: copper (Cu^2+^), chromium (Cr^3+^), cobalt (Co^2+^), manganese (Mn^2+^), nickel (Ni^2+^), zinc (Zn^2+^), iron (Fe^3+^), strontium (Sr^2+^), cadmium (Cd^2+^) and lead (Pb^2+^). The sources of ions were the following metal salts: CuSO_4_, Cr(NO_3_)_3_, Co(NO_3_)_2_, Mn(NO_3_)_2_, Ni(NO_3_)_2_, ZnSO_4_, Fe(NO_3_)_3_, Sr(NO_3_)_2_, Cd(NO_3_)_2_ and Pb(NO_3_)_2_. The salts were dissolved in deionized water. The tested variants of solutions contained only one tested metal ion in one concentration. Five sets of concentrations normalized to 25 mg L^−1^, 50 mg L^−1^, 100 mg L^−1^, 200 mg L^−1^ and 400 mg L^−1^ of each metal ion in the solution were prepared. The control was a plant grown on deionized water alone. The bulbs of *A. cepa* were grown in specific plastic containers filled with 30 mL solution. The research was carried out under hydroponic cultivation. Solutions were changed regularly to prevent contamination. The pH of the solutions was also controlled. Bulbs were grown under normal conditions of light (daily and night) at 21–25 °C and air humidity of 60%, over a three-week period.

### 4.2. Preparation of Samples for Determination

#### 4.2.1. Drying Procedure

The developed roots were rinsed with deionized water in order to remove of metal ions deposited on the outer parts of the roots. This procedure was intended to eliminate the possibility of contamination errors in the analysis. Each bulb was weighed using an analytical scale (Radwag, Poland). In order to avoid contaminants, to crush the raw material, a plastic knife was used that did not contain the tested metals. For the analysis, the roots, granary parts and assimilation leaves were separated. The drying of samples of plant raw material was carried out in a dryer (WAMED, Poland), choosing the appropriate working temperature of the device for the tested material. When a constant sample weight was obtained, the drying was finished. The results from three weight measurements did not differ from each other by more than 0.01 g. The crushed material was further analyzed.

#### 4.2.2. Mineralization Procedure

The prepared material was weighed on an analytical scale (Radwag, Radom, Poland) in an amount of about 0.5 g; for roots, this was about 0.15 g. The tested material was transferred to a Teflon cuvette; then, 1 mL of 69% (*v*/*v*) HNO_3_ (Carl Roth, Karlsruhe, Germany) and 9 mL of deionized water were added. The components were thoroughly mixed. The reaction vessel was closed and placed in a microwave mineralizer Multiwave 5000 (Anton Paar, Graz, Austria). The mineralizer worked in the following stages: I—pressure: 17–20 atm., power: 60% (5 min.), II—pressure: 30–33 atm., power: 80% (5 min.), III—pressure: 43–45 atm., power: 100% (10 min.). After these stages, the samples were cooled for 10 min. The solutions obtained after digestion were quantitatively transferred to calibrated sterile falcon tubes and adjusted to a final volume of 10 mL with deionized water.

### 4.3. Determination of Metal Ions

#### 4.3.1. Preparation of Solutions to Plot Calibration Curves

To calibrated flasks with a capacity of 100 mL, the appropriate amount of the standard solution of the selected metal at a concentration of 1000 mg L^−1^ (Merck, Darmstadt, Germany) was measured and adjusted with deionized water. The prepared standard solutions were used to obtain calibration curves on an atomic absorption spectrometry apparatus in the ASpect CS 2.0.0 software (Analytik Jena AG, Jena, Germany).

#### 4.3.2. F-AAS Conditions

The determination was carried out using the AAS method with flame atomization (F-AAS). The oxidizing gas was air and combustible acetylene. After atomization, the atoms of the elements passed to the measuring zone, where they absorbed radiation. The source of radiation was a high-pressure xenon arc lamp. The measurements were performed at λ = 228.80 nm for cadmium, λ = 240.73 nm for cobalt, λ = 324.75 nm for copper, λ = 359.35 nm for chromium, λ = 279.48 nm for manganese, λ = 232.00 nm for nickel, λ = 283.31 nm for lead, λ = 248.33 nm for iron, λ = 460.73 nm for strontium and λ = 213.84 nm for zinc. The measured quantity was the absorbance, whose value changed during the measurement, giving a signal in the form of a peak. Based on the measured absorbance values in relation to the calibration curve, the concentration [mg L^−1^] was determined and read using the specialized ASpect Cs 2.0.0 software (Analytik Jena AG, Germany). After taking into account the weight of the samples and the dilutions used, the ion content in individual parts of *A. cepa* was calculated. The content of each metal was converted to µg g^−1^ DW. The results were presented as the arithmetic mean ± SD of the obtained measurements.

### 4.4. Statistical Analysis

In the study, the organoleptic evaluation of growth trends and potential differences in elemental concentrations within onion bulbs based on varying nutrient levels was substantiated through rigorous statistical testing. Unfortunately, the classical assumptions of the analysis of variance (ANOVA) test, specifically the normality of the distribution of the dependent variable in subgroups and the homogeneity of variances, were not met.

The normality assumption was assessed using the Shapiro–Wilk test [56], and the assumption of the homogeneity of variances was tested using Levene’s test [57]. Due to the small sample sizes in the groups, normality was examined through the residuals of the ANOVA model, equivalent to checking normality in subgroups [58]. The analysis of residuals for each elemental concentration’s ANOVA model revealed a lack of normality in the majority of cases. Additionally, the hypothesis of homogeneity of variances was consistently rejected.

In order to standardize the methodology to test the significance of differences, a decision was made to employ the same test for each elemental analysis. The literature suggests that, in such situations, the Kruskal–Wallis (K-W) test is an appropriate choice [59]. However, given the small sample sizes and the low power of the K-W test, a non-parametric test based on resampling was proposed [60]. The most suitable choice was the bootstrap test proposed by Efron [61] due to its robustness and adaptability to small sample sizes.

Since tests for all elements and all three parts of the onion showed statistically significant differences, their results are not included in the text. Finally, post-hoc tests were performed to test the significance of differences between the different levels of supplementation. Since the 0 concentration level served as a quasi-control group, reflecting the natural condition for onion growth without supplementation, differences were determined by comparing each level to the control group using the Dunnett test [62].

All statistical analyses and visualizations were performed in the R statistical environment [63]. The following libraries of the R environment were used to perform individual tests and obtain graphs: rstatix [64], multcomp [65] and ggpubr [66].

## 5. Conclusions

The obtained results confirmed that *A. cepa* had a strong ability to bioaccumulate the tested heavy metals. The maximal accumulation of metal ions increased in the following order for *A. cepa* roots: Co^2+^ < Cr^3+^ < Fe^3+^ < Ni^2+^ < Sr^2+^ < Pb^2+^ < Cu^2+^ < Cd^2+^ < Zn^2+^ < Mn^2+^. For assimilation leaves, it was Pb^2+^ < Cr^3+^ < Cu^2+^ < Cd^2+^ < Fe^3+^ < Co^2+^ < Ni^2+^ < Zn^2+^ < Sr^2+^ < Mn^2+^; for bulbs, it was Cu^2+^ < Co^2+^ < Zn^2+^ < Sr^2+^ < Cr^3+^ < Fe^3+^ < Ni^2+^ < Cd^2+^ < Pb^2+^ < Mn^2+^.

The accumulation of metal ions was strongly dependent on their concentration in the solution and the analyzed part of the plant. The highest accumulation of metal ions was confirmed for the roots and ranged from 8.48 to 5912.34 µg g^−1^ DW. The assimilation leaves were characterized by highly varied metal ion content, from 0.70 to 1600.92 µg g^−1^ DW. The content of accumulated metal ions in the bulbs of *A. cepa* ranged from 0.60 to 186.38 µg g^−1^ DW. All parts of *A. cepa* were characterized by the high accumulation of Mn^2+^. The lowest accumulation was confirmed for Co^2+^ in the roots, Pb^2+^ in the assimilation leaves and Cu^2+^ in the bulbs of *A. cepa*.

In control samples grown in a solution without the tested metal ions, we confirmed the presence of the following metal ions: Cr^3+^, Cu^2+^, Fe^3+^, Mn^2+^ and Zn^2+^ for roots and bulbs; Mn^2+^ and Fe^3+^ for assimilation leaves. The maximal content of Cr^3+^, Cu^2+^, Fe^3+^, Mn^2+^ and Zn^2+^ in roots and bulbs grown in solutions containing the tested metal ions was, respectively, 21.5, 460.8, 81.5, 507.5 and 188.9 times higher for roots and 122.9, 7.6, 11.3, 154.0 and 35.5 times higher for bulbs than in control samples. The maximal content of Mn^2+^ and Fe^3+^ in assimilation leaves grown in solutions containing the tested metal ions was, respectively, 2501.4 and 7.1 times higher than in control samples.

Since our research concerned the bioaccumulation of heavy metals from individual solutions, it would be interesting to carry out further stages of research including the use of solutions containing mixtures of various heavy metals. Such experiments will allow us to analyze the synergistic and antagonistic relationships of these components in the bioaccumulation process.

## Figures and Tables

**Table 1:** Content of metal ions (µg g^−1^ DW ± SD) in roots of *A. cepa* depending on the concentration of metal ions in the solution (mg L^−1^).

Metal Ions	Min. Accumulation of Metal Ions (µg g^−1^ DW ± SD)	Concentration of Metal Ions in Solution (mg L^−1^)	Max. Accumulation of Metal Ions (µg g^−1^ DW ± SD)	Concentration of Metal Ions in Solution (mg L^−1^)	Control (µg g^−1^ DW ± SD)
Mn^2+^	3589.09 ± 29.57 ***	50	5912.34 ± 47.61 ***	100	11.65 ± 0.01
Ni^2+^	1057.85 ± 5.59 ***	50	2956.00 ± 43.84 ***	100	nd ^1^
Pb^2+^	805.10 ± 17.68 ***	25	3961.00 ± 35.36 ***	100	nd
Cd^2+^	5478.00 ± 22.63 ***	25	5478.00 ± 22.63 ***	25	nd
Zn^2+^	2015.34 ± 15.08 ***	25	5743.33 ± 0.00 ***	100	30.41 ± 1.32
Sr^2+^	8.48 ± 0.80	25	3727.27 ± 2.57 ***	400	nd
Cr^3+^	459.25 ± 0.35 ***	25	1008.42 ± 8.37 ***	50	47.02 ± 0.02
Fe^3+^	97.79 ± 3.69 *	100	1370.80 ± 41.01 ***	50	16.80 ± 0.15
Co^2+^	71.80 ± 0.10 ***	25	302.49 ± 2.36 ***	100	nd
Cu^2+^	1347.66 ± 14.62 ***	25	5013.33 ± 0.00 ***	100	10.88 ± 0.18

Notes: nd ^1^—not detected, the level of significance of post-hoc test is denoted by *—*p* < 0.05, ***—*p* < 0.001.

**Table 2:** Content of metal ions (µg g^−1^ DW ± SD) in assimilation leaves of *A. cepa* depending on the concentration of metal ions in the solution (mg L^−1^).

Metal Ions	Min. Accumulation of Metal Ions (µg g^−1^ DW ± SD)	Concentration of Metal Ions in Solution (mg L^−1^)	Max. Accumulation of Metal Ions (µg g^−1^ DW ± SD)	Concentration of Metal Ions in Solution (mg L^−1^)	Control (µg g^−1^ DW ± SD)
Mn^2+^	5.16 ± 0.13	25	1600.92 ± 1.29 ***	400	0.64 ± 0.01
Ni^2+^	27.94 ± 0.54 ***	50	224.70 ± 1.63 ***	100	nd ^1^
Pb^2+^	0.70 ± 0.04 **	50	1.17 ± 0.21 ***	25	nd
Cd^2+^	20.03 ± 4.03 ***	25	20.03 ± 4.03 ***	25	nd
Zn^2+^	62.34 ± 0.98 ***	25	457.06 ± 8.68 ***	100	nd
Sr^2+^	9.42 ± 0.09	25	626.64 ± 14.14 ***	400	nd
Cr^3+^	0.88 ± 0.04 ***	25	9.56 ± 0.04 ***	50	nd
Fe^3+^	5.46 ± 0.07	400	33.49 ± 1.06 ***	50	4.74 ± 0.03
Co^2+^	25.86 ± 0.69 ***	25	37.77 ± 0.32 ***	100	nd
Cu^2+^	4.74 ± 0.74 ***	25	14.28 ± 0.08 ***	100	nd

Notes: nd ^1^—not detected, the level of significance of post-hoc test is denoted by **—*p* < 0.01, ***—*p* < 0.001.

**Table 3:** Content of metal ions (µg g^−1^ DW ± SD) in the bulbs of *A. cepa* depending on the concentration of metal ions in the solution (mg L^−1^).

Metal Ions	Min. Accumulation of Metal Ions (µg g^−1^ DW ± SD)	Concentration of Metal Ions in Solution (mg L^−1^)	Max. Accumulation of Metal Ions (µg g^−1^ DW ± SD)	Concentration of Metal Ions in Solution (mg L^−1^)	Control (µg g^−1^ DW ± SD)
Mn^2+^	4.49 ± 0.48	25	186.38 ± 1.30 ***	400	1.21 ± 0.01
Ni^2+^	17.38 ± 1.73 ***	50	128.82 ± 1.56 ***	400	nd ^1^
Pb^2+^	2.40 ± 0.37 **	25	144.47 ± 1.09 ***	400	nd
Cd^2+^	0.60 ± 0.01	25	138.08 ± 21.13 ***	400	nd
Zn^2+^	39.21 ± 0.69 ***	25	53.97 ± 1.57 ***	400	1.52 ± 0.01
Sr^2+^	0.93 ± 0.13	25	55.07 ± 2.45 ***	400	nd
Cr^3+^	0.92 ± 0.11 *	25	66.37 ± 0.04 ***	400	0.54 ± 0.01
Fe^3+^	5.26 ± 1.12 **	25	98.25 ± 0.24 ***	400	8.70 ± 0.05
Co^2+^	7.51 ± 0.51 *	50	49.79 ± 3.66 ***	400	nd
Cu^2+^	1.38 ± 0.12	50	3.86 ± 0.33 ***	100	0.51 ± 0.10

Notes: nd ^1^—not detected, the level of significance of post-hoc test is denoted by *—*p* < 0.05, **—*p* < 0.01, ***—*p* < 0.001.

## Data Availability

Data are contained within the article.

## Supplementary Materials

The following supporting information can be downloaded at: https://www.mdpi.com/article/10.3390/molecules29010101/s1, Table S1: Comparison of average manganese content (µg g^−1^ DW) in the bulbs, roots and assimilation leaves of *A. cepa* depending on the concentration in the solution (mg L^−1^). Data are presented as mean ± SD; Table S2: Comparison of average nickel content (µg g^−1^ DW) in the bulbs, roots and assimilation leaves of *A. cepa* depending on the concentration in the solution (mg L^−1^). Data are presented as mean ± SD; Table S3: Comparison of average lead content (µg g^−1^ DW) in the bulbs, roots and assimilation leaves of *A. cepa* depending on the concentration in the solution (mg L^−1^). Data are presented as mean ± SD; Table S4: Comparison of average cadmium content (µg g^−1^ DW) in the bulbs, roots and assimilation leaves of *A. cepa* depending on the concentration in the solution (mg L^−1^). Data are presented as mean ± SD; Table S5: Comparison of average zinc content (µg g^−1^ DW) in the bulbs, roots and assimilation leaves of *A. cepa* depending on the concentration in the solution (mg L^−1^). Data are presented as mean ± SD; Table S6: Comparison of average strontium content (µg g^−1^ DW) in the bulbs, roots and assimilation leaves of *A. cepa* depending on the concentration in the solution (mg L^−1^). Data are presented as mean ± SD; Table S7: Comparison of average chromium content (µg g^−1^ DW) in the bulbs, roots and assimilation leaves of *A. cepa* depending on the concentration in the solution (mg L^−1^). Data are presented as mean ± SD; Table S8: Comparison of average iron content (µg g^−1^ DW) in the bulbs, roots and assimilation leaves of *A. cepa* depending on the concentration in the solution (mg L^−1^). Data are presented as mean ± SD; Table S9: Comparison of average cobalt content (µg g^−1^ DW) in the bulbs, roots and assimilation leaves of *A. cepa* depending on the concentration in the solution (mg L^−1^). Data are presented as mean ± SD; Table S10: Comparison of average copper content (µg g^−1^ DW) in the bulbs, roots and assimilation leaves of *A. cepa* depending on the concentration in the solution (mg L^−1^). Data are presented as mean ± SD.
