# Supplementary material for: Bioaccumulation Capacity of Onion (Allium cepa L.) Tested with Heavy Metals in Biofortification
Source: Molecules. 2023 Dec 22;29(1):101. doi: 10.3390/molecules29010101 (PMC10780257; doi:10.3390/molecules29010101)
Supplement: Supplementary file 1 [file molecules-29-00101-s001.zip › molecules-2768166-supplementary.pdf]

Supplementary Material

Bioaccumulation capacity of onion (*Allium cepa* L.) tested with heavy metals in biofortification

Katarzyna Czarnek [1,*], Małgorzata Tatarczak-Michalewska [2], Agnieszka Szopa [3], Marta Klimek-Szczykutowicz [4], Karolina Jafernik [3], Dariusz Majerek [5] and Eliza Blicharska [2,*]

Table S1. Comparison of average manganese content (μg g^{-1} DW) in the bulbs, roots and assimilation leaves of *A. cepa* depending on the concentration in the solution (mg L^{-1}). Data are presented as mean ± SD.

Part of the plant	Concentration of Mn^{2+} in the solution (mg L^{-1})					
	0	25	50	100	200	400
	Content of accumulated Mn^{2+} (μg g^{-1} DW±SD)					
Bulbs	1.21±0.01	4.49±0.48	31.94±0.32	61.10±0.88	147.54±3.28	186.38±1.30
Roots	11.65±0.01	3723.18±112.49	3589.09±29.57	5912.34±47.61	4075.00±23.58	3883.16±3.06
Assimilation leaves	0.64±0.01	5.16±0.13	51.33±3.96	256.32±4.68	521.36±6.43	1600.92±1.29

Table S2. Comparison of average nickel content (μg g^{-1} DW) in the bulbs, roots and assimilation leaves of *A. cepa* depending on the concentration in the solution (mg L^{-1}). Data are presented as mean ± SD.

Part of the plant	Concentration of Ni^{2+} in the solution (mg L^{-1})					
	0	25	50	100	200	400
	Content of accumulated Ni^{2+} (μg g^{-1} DW±SD)					
Bulbs	nd [1]	43.85±2.19	17.38±1.73	66.17±1.26	89.89±1.12	128.82±1.56
Roots	nd	2084.00±18.38	1057.85±5.59	2956.00±43.84	na [2]	na
Assimilation leaves	nd	30.22±0.06	27.94±0.54	224.70±1.63	na	na

[1] nd- not detected

[2] na- not analyzed

Table S3. Comparison of average lead content (μg g^{-1} DW) in the bulbs, roots and assimilation leaves of *A. cepa* depending on the concentration in the solution (mg L^{-1}). Data are presented as mean ± SD.

Part of the plant	Concentration of Pb^{2+} in the solution (mg L^{-1})					
	0	25	50	100	200	400
	Content of accumulated Pb^{2+} (μg g^{-1} DW±SD)					
Bulbs	nd [1]	2.40±0.37	5.46±0.03	7.19±0.08	8.38±0.36	144.47±1.09
Roots	nd	805.10±17.68	1313.80±7.92	3961.00±35.36	na [2]	na
Assimilation leaves	nd	1.17±0.21	0.70±0.04	0.92±0.01	na	na

[1] nd- not detected

[2] na- not analyzed

Table S4. Comparison of average cadmium content (μg g^{-1} DW) in the bulbs, roots and assimilation leaves of *A. cepa* depending on the concentration in the solution (mg L^{-1}). Data are presented as mean ± SD.

Part of the plant	Concentration of Cd^{2+} in the solution (mg L^{-1})					
	0	25	50	100	200	400
	Content of accumulated Cd^{2+} (μg g^{-1} DW±SD)					
Bulbs	nd [1]	0.60±0.01	1.21±0.09	2.86±0.16	50.36±0.82	138.08±21.13
Roots	nd	5478.00±22.63	na [2]	na	na	na
Assimilation leaves	nd	20.03±4.03	na	na	na	na

[1] nd- not detected

[2] na- not analyzed

Table S5. Comparison of average zinc content (μg g^{-1} DW) in the bulbs, roots and assimilation leaves of *A. cepa* depending on the concentration in the solution (mg L^{-1}). Data are presented as mean ± SD.

Part of the plant	Concentration of Zn^{2+} in the solution (mg L^{-1})					
	0	25	50	100	200	400
	Content of accumulated Zn^{2+} (μg g^{-1} DW±SD)					
Bulbs	1.52±0.01	39.21±0.69	44.22±0.85	49.13±0.41	51.75±0.16	53.97±1.57
Roots	30.41±1.32	2015.34±15.08	3269.68±24.04	5743.33±0.00	na [2]	na
Assimilation leaves	nd [1]	62.34±0.98	134.50±1.17	457.06±8.68	na	na

[1] nd- not detected

[2] na- not analyzed

Table S6. Comparison of average strontium content (μg g^{-1} DW) in the bulbs, roots and assimilation leaves of *A. cepa* depending on the concentration in the solution (mg L^{-1}). Data are presented as mean ± SD.

Part of the plant	Concentration of Sr^{2+} in the solution (mg L^{-1})					
	0	25	50	100	200	400
	Content of accumulated Sr^{2+} (μg g^{-1} DW±SD)					
Bulbs	nd [1]	0.93±0.13	3.03±0.65	7.87±0.01	14.50±0.57	55.07±2.45
Roots	nd	8.48±0.80	573.14±4.56	768.50±0.83	1668.64±8.36	3727.27±2.57
Assimilation leaves	nd	9.41±0.09	17.78±0.74	18.70±0.42	151.64±0.52	626.64±14.14

[1] nd- not detected

Table S7. Comparison of average chromium content (μg g^{-1} DW) in the bulbs, roots and assimilation leaves of *A. cepa* depending on the concentration in the solution (mg L^{-1}). Data are presented as mean ± SD.

Part of the plant	Concentration of Cr^{3+} in the solution (mg L^{-1})					
	0	25	50	100	200	400
	Content of accumulated Cr^{3+} (μg g^{-1} DW±SD)					
Bulbs	0.54±0.01	0.92±0.11	1.83±0.07	3.24±0.17	8.19±0.01	66.37±0.04
Roots	47.02±0.02	459.25±0.35	1008.42±8.37	938.01±3.52	na	na
Assimilation leaves	nd [1]	0.88±0.04	9.56±0.04	na [2]	na	na

[1] nd- not detected

[2] na- not analyzed

Table S8. Comparison of average iron content (μg g^{-1} DW) in the bulbs, roots and assimilation leaves of *A. cepa* depending on the concentration in the solution (mg L^{-1}). Data are presented as mean ± SD.

Part of the plant	Concentration of Fe^{3+} in the solution (mg L^{-1})					
	0	25	50	100	200	400
	Content of accumulated Fe^{3+} (μg g^{-1} DW±SD)					
Bulbs	8.70±0.05	5.26±1.12	10.32±0.42	71.04±0.54	9.64±0.15	98.25±0.24
Roots	16.80±0.15	306.70±0.14	1370.80±41.01	97.79±3.69	212.80±0.28	577.30±2.69
Assimilation leaves	4.74±0.03	15.40±0.07	33.49±1.06	30.39±0.27	15.28±0.29	5.46±0.35

Table S9. Comparison of average cobalt content (μg g^{-1} DW) in the bulbs, roots and assimilation leaves of *A. cepa* depending on the concentration in the solution (mg L^{-1}). Data are presented as mean ± SD.

Part of the plant	Concentration of Co^{2+} in the solution (mg L^{-1})					
	0	25	50	100	200	400
	Content of accumulated Co^{2+} (μg g^{-1} DW±SD)					
Bulbs	nd [1]	10.54±0.01	7.51±0.51	15.04±0.26	29.88±2.60	49.79±3.66
Roots	nd	71.80±0.10	205.20±2.55	302.49±2.36	na [2]	na
Assimilation leaves	nd	25.86±0.69	29.70±1.28	37.77±0.32	na	na

[1] nd- not detected

[2] na- not analyzed

Table S10. Comparison of average copper content (μg g^{-1} DW) in the bulbs, roots and assimilation leaves of *A. cepa* depending on the concentration in the solution (mg L^{-1}). Data are presented as mean ± SD.

Part of the plant	Concentration of Cu^{2+} in the solution (mg L^{-1})					
	0	25	50	100	200	400
	Content of accumulated Cu^{2+} (μg g^{-1} DW±SD)					
Bulbs	0.51±0.10	2.22±0.08	1.38±0.12	3.86±0.33	1.82±0.31	2.26±0.90
Roots	10.88±0.18	1347.66±14.62	3420.34±8.01	5013.33±0.00	na [2]	na
Assimilation leaves	nd [1]	4.74±0.74	5.73±0.01	14.28±0.08	na	na

[1] nd- not detected

[2] na- not analyzed
